# Development and validation of a prognostic nomogram for predicting cancer‐specific survival after radical cystectomy in patients with bladder cancer:A population‐based study

**DOI:** 10.1002/cam4.3535

**Published:** 2020-10-16

**Authors:** Zhiqiang Yang, Yunjin Bai, Maoying Liu, Xu Hu, Ping Han

**Affiliations:** ^1^ Department of Urology West China Hospital Sichuan University Chengdu People's Republic of China; ^2^ West China School of Medicine/West China Hospital Sichuan University Chengdu People's Republic of China; ^3^ Anyue Hengkang Hospital Anyue County People's Republic of China

**Keywords:** bladder cancer, nomogram, prognosis, radical cystectomy, SEER database

## Abstract

**Purpose:**

To establish a prognostic model to estimate the cancer‐specific survival (CSS) for urothelial carcinoma of bladder (UCB) patients after radical cystectomy (RC).

**Methods:**

A total of 8650 candidates (2004–2011) obtained from the Surveillance, Epidemiology, and End Results (SEER) database were randomly split into development cohort (*n* = 4323) and validation cohort (*n* = 4327). We performed Cox regression analysis to identify prognostic factors and Kaplan‐Meier analysis to assess survival outcome. A nomogram predicting CSS was constructed. Its performance was validated by calibration curves, the receiver operating characteristic (ROC) curves, concordance index (C‐index), decision curve analysis (DCA), the net reclassification improvement (NRI), and the integrated discrimination improvement (IDI).

**Results:**

The nomogram incorporated marital status, T stage, N stage, tumor size, and chemotherapy. In validation cohort, C‐index of the nomogram was 0.707. AUC of the nomogram and AJCC stage were 0.767 versus 0.674. Calibration plots for 3‐ and 5‐year CSS displayed good concordance. DCA curves of the nomogram exhibited larger benefits than the AJCC stage. The NRI and IDI indicated the nomogram outperformed AJCC stage.

**Conclusions:**

We have established a prognostic nomogram with improved discriminative ability and clinical benefits for UCB patients after RC. The nomogram alongside an easy access web tool may assist clinicians in optimizing the postoperative management.

## Graphical abstract

This population‐based study has established a nomogram and its derived webserver to predict cancer‐specific survival probabilities for patients with urothelial carcinoma of the bladder after radical cystectomy. The model’s discriminative abilities, accuracy and clinical benefits were validated and performed better than traditional AJCC stage.

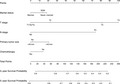

## 1 INTRODUCTION

Urothelial carcinoma of the bladder (UCB) is the 12th most common malignancy globally, with nearly 550,000 new cases and 200,000 deaths each year.^1^ Radical cystectomy (RC) with pelvic lymph node dissection is the standard treatment of muscle‐invasive UCB or high‐risk non‐muscle‐invasive UCB, with or without chemotherapy.^2^, ^3^

To predict clinical and oncologic outcomes after RC is essential, as those with poor estimated survival might be candidates for and benefit from adjuvant therapies or potential clinical trials. The prognostic roles of various factors and predictive models for UCB have been studied for years. Traditionally, clinicians rely on the American Joint Committee of Cancer (AJCC) stage guidelines which comprise primary tumor (T) status, regional lymph node (N), and distant metastasis (M).^4^ However, AJCC staging system does not consider demographic factors or treatment modalities, which also contribute significantly to outcome prediction. For instance, a previous study found that age above 80 years old was independently associated with higher risk of recurrence and worse clinical outcomes after RC.^5^ Besides, pelvic lymph node dissection results were reported to predict survival outcomes for patients undergoing RC more accurately than the traditional N stage.^6^, ^7^ Given that predicted outcomes based solely on AJCC stages have displayed relatively low accuracy and significant heterogeneity for individual patients, other prognostic models have been put forward.^8^

Nomograms are now one of the most widely used prediction tools that provide tailored individual prognostic information by incorporating significant demographic, clinical, pathological or treatment features, and presenting simple visualized results of statistical analysis.^9^ Nomograms that predict survival after RC have been previously developed and externally validated.^10^, ^11^, ^12^, ^13^ Although these models showed acceptable accuracy, their application has been limited because some of the included variables are not generally available and calculation of the results requires heavy endeavor. Moreover, the AJCC N stage used in these nomograms has displayed limited prognostic value.^14^ An applicable prognostic tool should not only consider significantly predictive and easily available variables but also simplify the usage of it.

In this study, we aim to establish a novel prognostic nomogram to assess relevant prognostic factors and estimate the cancer‐specific survival for patients after RC. Our study is based on a large population derived from the Surveillance, Epidemiology, and End Results (SEER) database. In addition, we demonstrated the nomogram's discriminative ability and clinical practicality by comparing it with the AJCC stage. We then developed a novel web tool for easy access of our model and improved counseling of patients after RC.

## 2 MATERIALS AND METHODS

### 2.1 Patient selection

Patient records were retrieved from the SEER database by SEER*Stat version 8.3.6. SEER contains cancer incidence data collected by 18 population‐based cancer registries which cover nearly 35% U.S. population. The inclusion criteria were as follows: (a) bladder cancer cases diagnosed from 2004 to 2011; (b) patients who received RC and lymph node dissection; (c) age not under 18 years old; (d) with clear classification of races; (e) with complete information of AJCC stages and tumor‐node‐metastasis (TNM) stages; (f) with clear marital status; (g) with clear racial information; (h) with definitive survival duration or follow‐up time. Patients with distant metastasis (M1) or missing information of any of above conditions were excluded from this study. Since SEER is a publicly available database and all records have been de‐identified, no additional ethical approval or informed consent was required after the SEER Research Data Agreement was signed for accessing data.

### 2.2 Variable selection

This study collected information of variables including age at diagnosis, gender, race, marital status, year of diagnosis, histologic subtype, histologic grade, AJCC stage, T stage, N stage, radiotherapy, chemotherapy, primary tumor size, SEER cause‐specific death classification, vital status, and survival time (months). In SEER database, records between 2004 and 2011 were coded with the sixth edition of AJCC stages. Age was a numeric factor and for application simplicity, it was converted into categorical form according to real‐world experience and previous studies.^15^ Marital status was defined as married, seperatied, divorced or widowed (SDW), or never‐married. Histologic grade was categorized by G1–G2 (low‐grade) and G3–G4 (high‐grade), considering low‐grade patients were too few to be subdivided. When it came to primary tumor size, we adopted its median as the cutoff value. Cancer‐specific survival (CSS), which was identified by SEER cause specific death classification and survival months, was adopted as primary outcome.

### 2.3 Statistical analyses

The total records were randomly split into development and validation cohorts in a ratio of 1:1. The univariate Cox proportional hazards regression model was applied in the development cohort for estimating the hazard ratio (HR) and corresponding 95% confidence interval (CI) to identify potential significant prognostic factors. The factors were then incorporated into multivariate analytic model to determine their independent association with CSS in the same cohort. Survival analyses were performed by the Kaplan–Meier method and log‐rank tests were used to estimate the differences of CSS stratified by each factor. Based on final screened variables, the nomogram was constructed for visualized prediction of 3‐ and 5‐year survival probability of development cohort. In addition, a web tool was created using the shiny package of R 3.6.1 for easy access and convenient application of this model.

The internal validation of the nomogram was conducted in development cohort and the external validation was performed using validation cohort. The discriminative ability of the nomogram was assessed by the concordance index (C‐index) and the receiver operating characteristic (ROC) curves with the calculated area under the curve (AUC). Calibration plots were employed for comparing nomogram‐predicted and actual outcomes of 3‐ and 5‐year survival time. Both discrimination analyses and calibration plots used bootstrapping with 500 resamples. In addition, decision curve analysis (DCA) was applied to estimate the clinical usefulness and benefits of the nomogram by comparing the threshold probabilities range of the model to that of the AJCC staging system.^16^ Besides, for comparing the accuracy of the model with that of the AJCC stage, the net reclassification improvement (NRI) and the integrated discrimination improvement (IDI) were evaluated.^17^ *Z* test was used to assess the differences.

All statistical analyses were conducted by R version 3.6.1 (http://www.R‐project.org, The R Foundation for Statistical Computing) via RStudio software version 1.2.5033 and EmpowerStats (http://www.empowerstats.com, X&Y Solutions, Inc.). A *p* < 0.05 was considered statistically significant.

## 3 RESULTS

### 3.1 Patient characteristics

**Figure 1:**
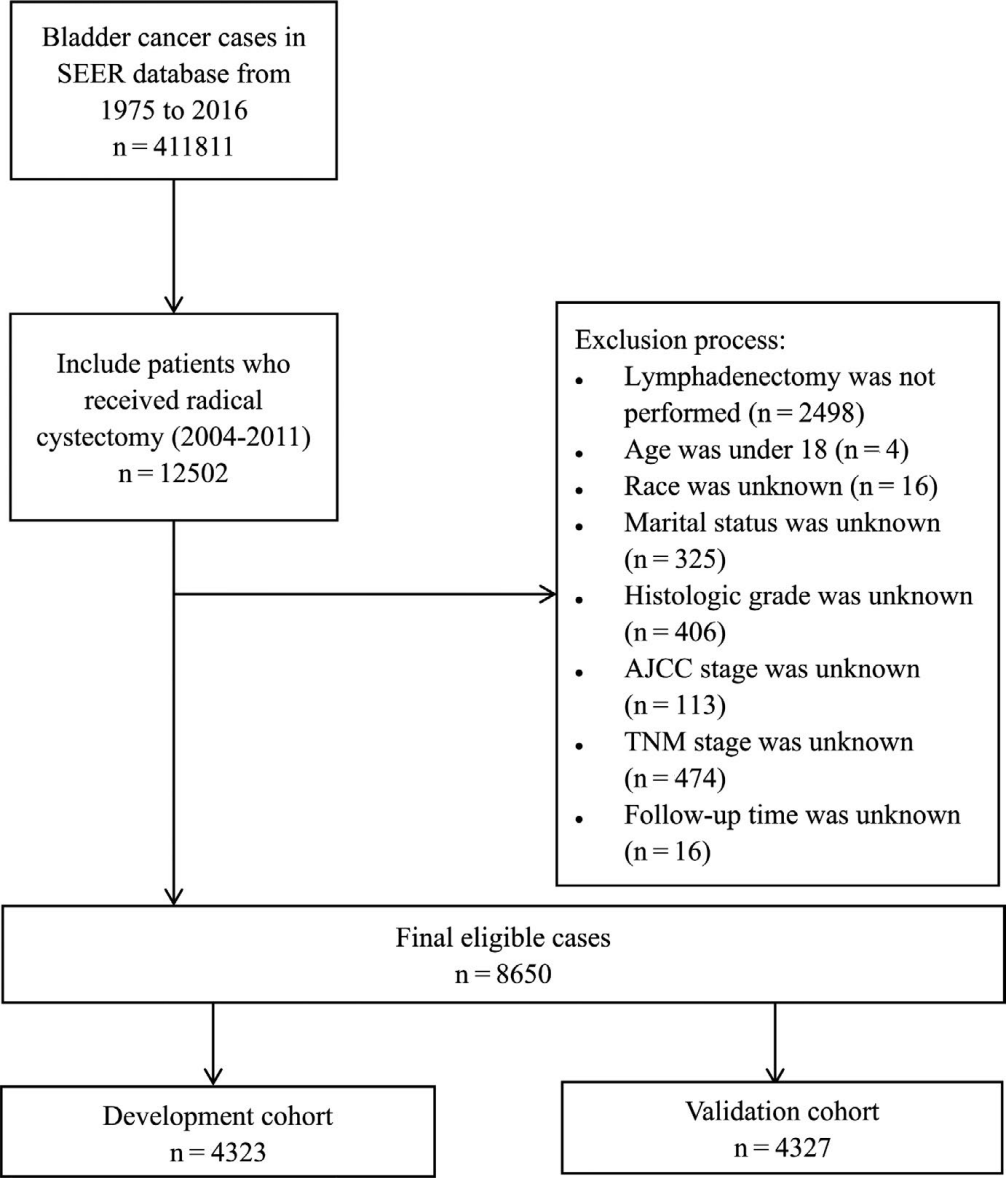
Flow diagram of filtering and selecting patient records from the SEER database. SEER: Surveillance, Epidemiology, and End Results. AJCC, American Joint Committee on Cancer; T, primary tumor; N, regional lymph node; M: metastasis

A total of 8650 M0 patients who underwent RC and lymph node dissection were retrieved and screened for further analyses according to the inclusion criteria. They were randomly assigned into development cohort (*n* = 4323) and validation cohort (*n* = 4327) in a ratio of 1:1 (Figure 1). In total, there were 1428 (33.0%) UCB‐related deaths in the development cohort and 1415 (32.7%) UCB‐related deaths in the validation cohort. The 3‐ and 5‐year CSS rates were 71.3% and 65.9%, respectively, in the development cohort and 71.2% and 65.7%, respectively, in the validation cohort. Detailed demographic information and clinical characteristics of the two cohorts alongside their comparison are presented in Table 1. Patients characteristics include age (<60, 60–69, 70–79, >80), gender (Male, Female), race (White, Black, Other), marital status (Married, SDW, Never‐married), year of diagnosis (2004–2011), histology (Urothelial carcinoma, Non‐urothelial carcinoma), grade (G1–G2, G3–G4), AJCC stage (I/0a/0is, II, III, IV), T stage (T1/Ta/Tis, T2, T3, T4), N stage (N0, N+), radiotherapy (No, Yes), chemotherapy (No, Yes), and primary tumor size (<40 mm, >40 mm, Unknown). Median follow‐up time is 51 months in the development cohort and 50 months in the validation cohort (*p* = 0.176). Differences between the two cohorts in each variable are statistically insignificant (*p* > 0.05).

**Table 1:** Baseline demographical and clinicopathological characteristics of patients

Characteristics	Total cohort *N* (%)	Development cohort *N* (%)	Validation cohort *N* (%)	*p* value
Number of patients	8650	4323	4327	
Median age (25th–75th percentile)	68 (60–75)	68 (60–75)	68 (60–75)	0.665
Age				0.786
<60	1963 (22.7%)	978 (22.6%)	985 (22.8%)	
60–69	2759 (31.9%)	1365 (31.6%)	1394 (32.2%)	
70–79	2854 (33.0%)	1429 (33.1%)	1425 (32.9%)	
>80	1074 (12.4%)	551 (12.7%)	523 (12.1%)	
Gender				0.842
Male	6511 (75.3%)	3250 (75.2%)	3261 (75.4%)	
Female	2139 (24.7%)	1073 (24.8%)	1066 (24.6%)	
Race				0.335
White	7716 (89.2%)	3875 (89.6%)	3841 (88.8%)	
Black	508 (5.9%)	249 (5.8%)	259 (6.0%)	
Other	426 (4.9%)	199 (4.6%)	227 (5.2%)	
Marital status				0.658
Married	5765 (66.6%)	2862 (66.2%)	2903 (67.1%)	
SDW	1936 (22.4%)	977 (22.6%)	959 (22.2%)	
Never‐married	949 (11.0%)	484 (11.2%)	465 (10.7%)	
Year of diagnosis				0.653
2004	979 (11.3%)	505 (11.7%)	474 (11.0%)	
2005	970 (11.2%)	474 (11.0%)	496 (11.5%)	
2006	1043 (12.1%)	503 (11.6%)	540 (12.5%)	
2007	1143 (13.2%)	595 (13.8%)	548 (12.7%)	
2008	1123 (13.0%)	555 (12.8%)	568 (13.1%)	
2009	1087 (12.6%)	542 (12.5%)	545 (12.6%)	
2010	1203 (13.9%)	595 (13.8%)	608 (14.1%)	
2011	1102 (12.7%)	554 (12.8%)	548 (12.7%)	
Histology				0.200
Urothelial carcinoma	7874 (91.3%)	3962 (91.6%)	3932 (90.9%)	
Non‐urothelial carcinoma	756 (8.7%)	361 (8.4%)	395 (9.1%)	
Grade				0.744
G1–G2	523 (6.0%)	265 (6.1%)	258 (6.0%)	
G3–G4	8127 (94.0%)	4058 (93.9%)	4069 (94.0%)	
AJCC stage				0.671
I/0a/0is	917 (10.6%)	464 (10.7%)	453 (10.5%)	
II	2777 (32.1%)	1408 (32.6%)	1369 (31.6%)	
III	2500 (28.9%)	1227 (28.4%)	1273 (29.4%)	
IV	2456 (28.4%)	1224 (28.3%)	1232 (28.5%)	
T stage				0.800
T1/Ta/Tis	964 (11.1%)	490 (11.3%)	474 (11.0%)	
T2	3237 (37.4%)	1627 (37.6%)	1610 (37.2%)	
T3	2937 (34.0%)	1447 (33.5%)	1490 (34.4%)	
T4	1512 (17.5%)	759 (17.6%)	753 (17.4%)	
N stage				0.811
N0	6293 (72.8%)	3150 (72.9%)	3143 (72.6%)	
N+	2357 (27.2%)	1173 (27.1%)	1184 (27.4%)	
Radiotherapy				0.306
No	8369 (96.8%)	4191 (96.9%)	4178 (96.6%)	
Yes	281 (3.2%)	132 (3.1%)	149 (3.4%)	
Chemotherapy				0.347
No	2985 (34.5%)	1471 (34.0%)	1514 (35.0%)	
Yes	5665 (65.5%)	2852 (66.0%)	2813 (65.0%)	
Median primary tumor size (25th−75th percentile)	40 (25–55)	40 (25–55)	40 (25–55)	0.422
Primary tumor size				0.336
<40 mm	3060 (35.4%)	1556 (36.0%)	1504 (34.8%)	
>40 mm	3351 (38.7%)	1643 (38.0%)	1708 (39.5%)	
Unknown	2239 (25.9%)	1124 (26.0%)	1115 (25.8%)	
Median follow‐up months (25th–75th percentile)	50 (15–90)	51 (16–92)	50 (14–89)	0.176

Abbreviations: AJCC, American Joint Committee on Cancer; N, node; SDW, separated, divorced or widowed; T, tumor.

### 3.2 Identification of prognostic factors

**Figure 2:**
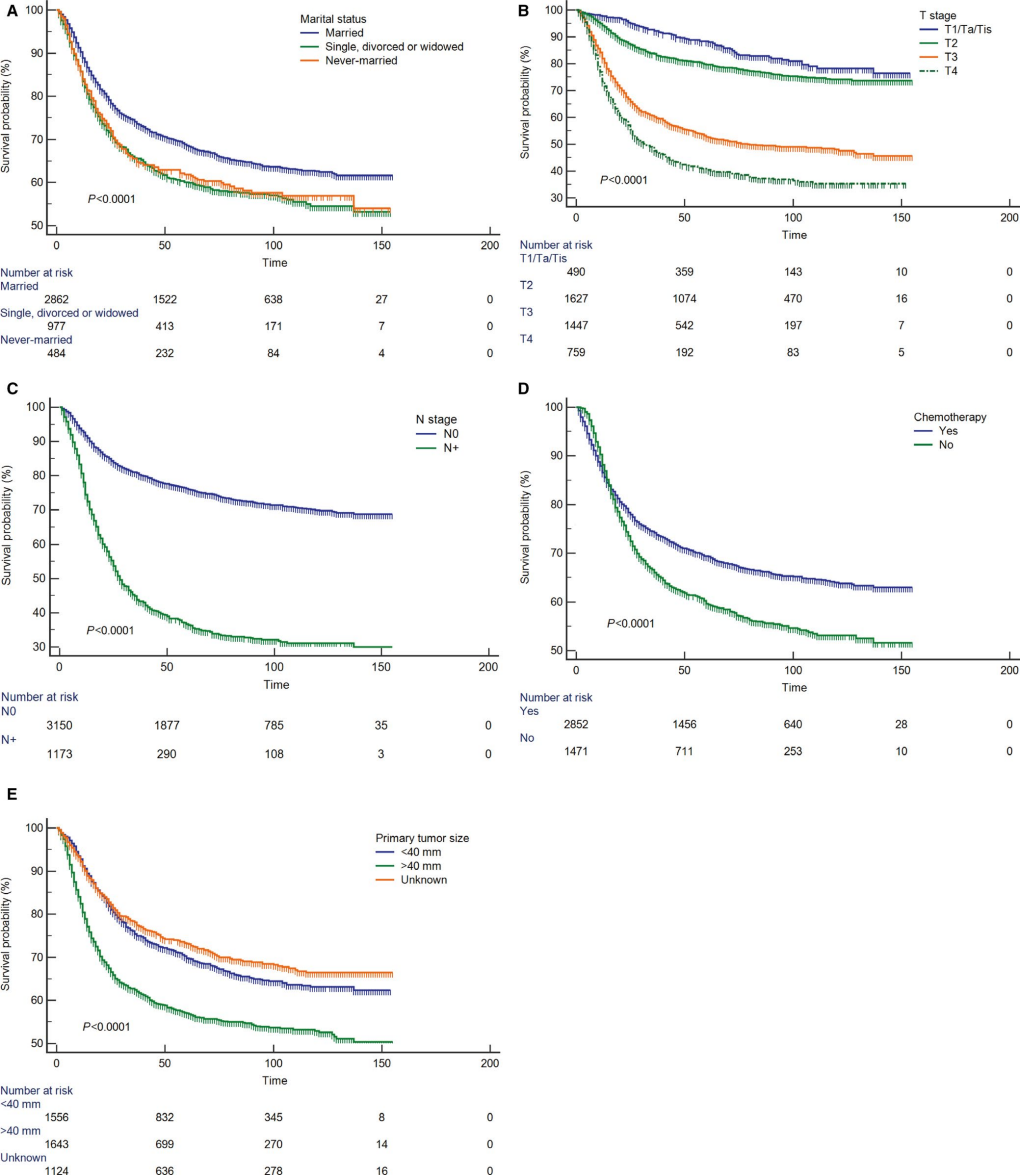
Kaplan‐Meier plots for describing cancer‐specific survival in patients after radical cystectomy stratified according to: (A) marital status (married vs. separated, divorced or widowed vs. never‐married); (B) T stage (T1/Ta/Tis vs. T2 vs. T3 vs. T4); (C) N stage (N0 vs. N+); (D) chemotherapy (No vs. Yes); (E) primary tumor size (<40 mm vs. >40 mm vs. unknown)

The univariate Cox proportional hazards regression analysis identified nine factors which were associated with CSS in the development cohort. Sequential multivariate Cox regression analysis incorporating these variables was performed to finally determine five independent prognostic factors as following: marital status, T stage, N stage, chemotherapy, and primary tumor size (Table 2). To name a few, SDW (HR = 1.2, 95% CI: 1.0–1.3, *p* = 0.017), never‐married status (HR = 1.2, 95% CI: 1.0–1.4, *p* = 0.012), higher T stage (T4 HR = 3.5, 95% CI: 2.7–4.5, *p* < 0.001), lymph node metastasis (HR = 2.6, 95% CI: 2.3–2.9, *p* < 0.001), and larger primary tumor size (HR = 1.3, 95% CI: 1.1–1.4, *p* < 0.001) were associated with worse CSS. While the administration of chemotherapy appeared to be a protective factor (HR = 0.8, 95% CI: 0.7–0.9, *p* = 0.001). Kaplan–Meier analyses demonstrate different survival outcomes stratified by each variable (Figure 2A–E). Log‐rank tests showed the differences among subgroups were statistically significant (*p* < 0.0001), indicating that factor stratifications were appropriate and acceptable.

**Table 2:** Univariate and multivariate Cox regression analyses of selected variables for cancer‐specific survival in the development cohort

Characteristics	Univariate analysis		Multivariate analysis	
	HR (95% CI)	*p* value	HR (95% CI)	*p* value
Age				
<60	Reference			
60–69	1.0 (0.8, 1.1)	0.607		
70–79	1.1 (0.9, 1.2)	0.242		
>80	1.0 (0.8, 1.2)	0.990		
Gender				
Male	Reference		Reference	
Female	1.2 (1.1, 1.3)	0.002*	1.1 (0.9, 1.2)	0.392
Race				
White	Reference		Reference	
Black	1.4 (1.1, 1.7)	0.002*	1.2 (1.0, 1.5)	0.077
Other	1.0 (0.8, 1.3)	0.926	0.9 (0.7, 1.2)	0.664
Marital status				
Married	Reference		Reference	
SDW	1.3 (1.2, 1.5)	<0.001*	1.2 (1.0, 1.3)	0.017*
Never‐married	1.3 (1.1, 1.5)	0.002*	1.2 (1.0, 1.4)	0.012*
Year of diagnosis				
2004	Reference			
2005	1.0 (0.8, 1.2)	0.832		
2006	1.0 (0.8, 1.2)	0.763		
2007	1.0 (0.8, 1.3)	0.806		
2008	1.0 (0.8, 1.2)	0.847		
2009	1.0 (0.8, 1.3)	0.693		
2010	0.9 (0.8, 1.2)	0.537		
2011	0.9 (0.7, 1.1)	0.384		
Histology				
Urothelial carcinoma	Reference		Reference	
Non‐urothelial carcinoma	1.4 (1.2, 1.7)	<0.001*	1.2 (1.0, 1.4)	0.098
Grade				
G1–G2	Reference			
G3–G4	1.1 (0.9, 1.4)	0.379		
T stage				
T1/Ta/Tis	Reference		Reference	
T2	1.4 (1.1, 1.8)	0.004*	1.3 (1.0, 1.7)	0.030*
T3	3.9 (3.1, 4.9)	<0.001*	2.8 (2.2, 3.5)	<0.001*
T4	5.5 (4.3, 7.1)	<0.001*	3.5 (2.7, 4.5)	<0.001*
N stage				
N0	Reference		Reference	
N+	3.5 (3.1, 3.8)	<0.001*	2.6 (2.3, 2.9)	<0.001*
Radiotherapy				
No	Reference		Reference	
Yes	2.2 (1.7, 2.7)	<0.001*	1.2 (1.0, 1.6)	0.106
Chemotherapy				
No	Reference		Reference	
Yes	0.7 (0.5, 0.9)	<0.001*	0.8 (0.7, 0.9)	0.001*
Primary tumor size				
<40 mm	Reference		Reference	
>40 mm	1.6 (1.4, 1.8)	<0.001*	1.3 (1.1, 1.4)	<0.001*
Unknown	0.9 (0.8, 1.0)	0.154	1.0 (0.9, 1.2)	0.870

Abbreviations: HR, hazard ratio; N, node; SDW, separated, divorced or widowed; T, tumor.

* *p* < 0.05, indicating statistical significance.

### 3.3 Development of a prognostic nomogram

**Figure 3:**
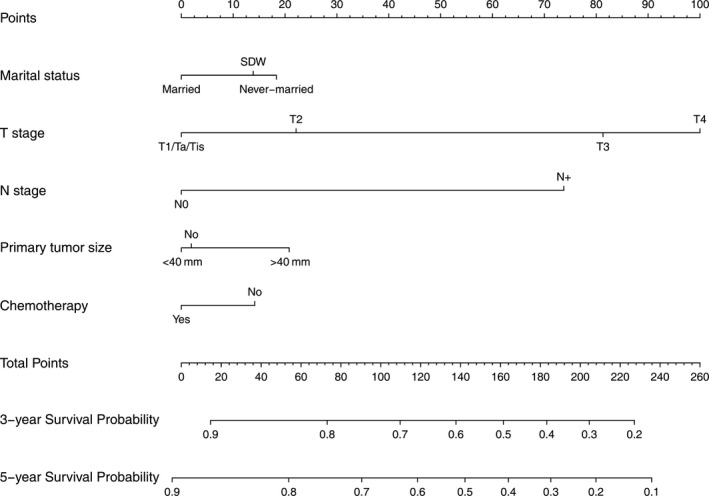
Nomogram predicting 3‐ and 5‐year bladder cancer‐specific survival probability for patients after radical cystectomy. Variables include marital status, T stage, N stage, primary tumor size, and chemotherapy

The prognostic nomogram predicting 3‐ and 5‐year bladder CSS probability was established based on the screened factors using 4323 patients from the development cohort (Figure 3). As demonstrated in the nomogram, T stage contributed most for the prognosis of CSS, followed by N stage, primary tumor size, marital status, and chemotherapy.

### 3.4 Validation of the nomogram

**Figure 4:**
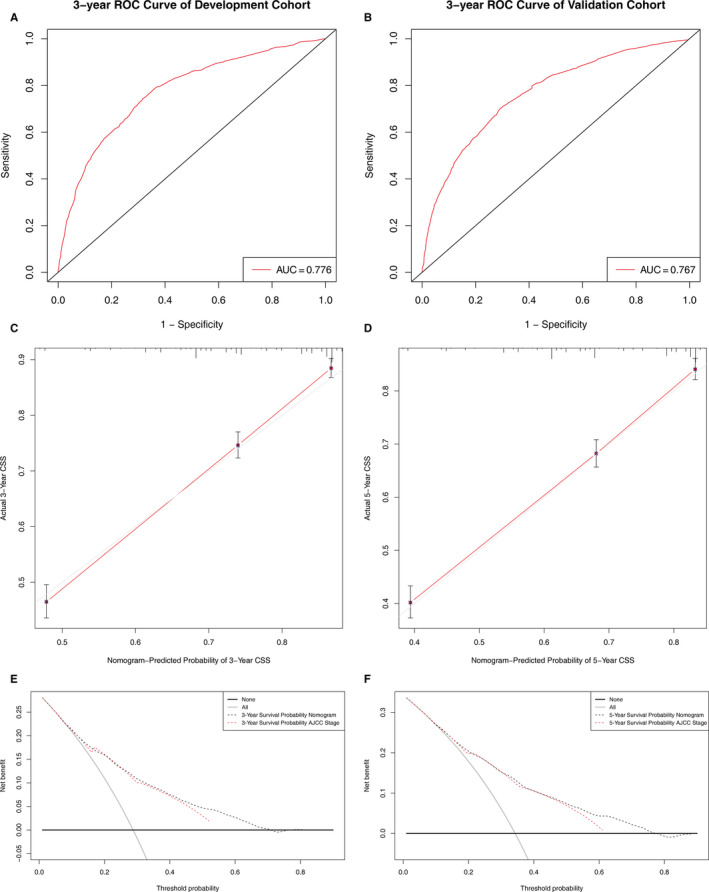
External validation of the nomogram: ROC curves of the nomogram predicting 3‐year CSS of the development cohort (A) and the validation cohort (B). Calibration plots of the nomogram describing 3‐ (C) and 5‐year (D) CSS. DCA curves comparing nomogram and AJCC stage in 3‐ (E) and 5‐year (F) scales. ROC, receiver operating characteristic; CSS, cancer‐specific survival; DCA, decision curve analysis; AJCC, American Joint Committee for Cancer

The nomogram for CSS was validated both externally and internally. The C‐index of the nomogram was 0.718 in the development cohort and 0.707 in the validation cohort, both significantly higher than that of the AJCC stage (*p* < 0.05), which was 0.695 and 0.683, respectively. Discriminative ability of the nomogram was also examined by ROC curves (Figure 4A,B). AUC of the nomogram was higher compared with the AJCC stage both in the development cohort (3‐year AUC: 0.776 vs. 0.703) and validation cohort (3‐year AUC: 0.767 vs. 0.674). Meanwhile, the calibration plots of development and validation cohorts for 3‐ and 5‐year CSS all demonstrated fine compliance between actual observations and predicted outcomes judged by eye (Figure 4C,D).

In addition, we compared the clinical benefits of the nomogram to that of AJCC staging group by performing DCA. As displayed in Figure 4E,F, the nomogram's DCA curves showed larger net benefits across a wide range of threshold probability than the AJCC stage model both for 3‐year and 5‐year CSS in the validation cohort, indicating this nomogram possesses better clinical usefulness.

Accuracy analysis showed that the NRI for 3‐ and 5‐year survival time were 0.202 (95% CI: 0.143–0.294) and 0.180 (95% CI: 0.128–0.269), respectively, in the validation cohort and 0.187 (95% CI: 0.139–0.285) and 0.165 (95% CI: 0.088–0.257), respectively, in the development cohort. Furthermore, the IDI for 3‐ and 5‐year survival were 1.5% (*p* < 0.001) and 1.4% (*p* < 0.001), respectively, in the validation cohort and 1.7% (*p* < 0.001) and 1.6% (*p* < 0.001), respectively, in the development cohort. These results suggest that the nomogram has greater potential to accurately predict prognosis compared with traditional AJCC staging system.

### 3.5 Development of a web tool for easy application of our model

An online web tool based on our nomogram can be accessed at https://prediction‐calculator.shinyapps.io/DynNomapp/. To utilize this tool, researchers and clinicians can simply input clinical and demographic features. Then, the output figures and tables generated by the tool can be read to directly learn the predicted survival probability across different time.

## 4 DISCUSSION

UCB is one of the major health issues across the globe, with both high morbidity and mortality. Although various treatment modalities have been developed, RC remains the standard therapy of muscle‐invasive UCB and high‐risk non‐muscle‐invasive UCB.^2^, ^3^ It is necessary to build predictive models to facilitate subsequent counseling, follow‐up scheduling, and clinical trial enrollment on individual levels for patients after RC. In this study, we established and validated a predictive nomogram based on several independent factors concerning demographic, clinical, pathologic, and treatment characteristics for individual prognosis using a large population of UCB patients who underwent RC.

Our study also assessed the clinical value of constructed nomogram by comparing it with traditional AJCC staging system. The nomogram outperforms AJCC stage both in the development and validation cohorts. In detail, our prognostic model shows better discriminative ability and accuracy for predicting 3‐ and 5‐year survival probabilities with higher C‐indices and AUCs, positive NRI and IDI. Besides, DCA curves demonstrate that our nomogram possesses better potential of clinical utility than the AJCC stage.

The nomogram for CSS probability prediction incorporated five factors including marital status, T stage, N stage, primary tumor size, and chemotherapy. Notably, to reduce selection bias and maintain the completeness of data, patients with unknown tumor size were reserved in our analysis. Each finally included variable was independently associated with survival outcomes of UCB patients, which was consistent with prior studies. Sammon et al. found that being married was an indicator of lower all‐cause mortality for both men and women after RC, compared with their SDW or never‐married counterparts.^18^ Higher T stage and lymph node metastasis have always been unfavorable factors for UCB patients.^2^, ^3^ Meanwhile, the administration of chemotherapy was reported to contribute to decreased cancer‐specific and overall mortality with no increased risk of perioperative morbidity after RC.^19^, ^20^ Besides, tumor size was also related with distinct outcomes.^21^, ^22^ But the optimal cutoffs of primary tumor size might not be universal due to relevant small population size. In short, the variables used to create the prognostic model in this study are statistically reliable in our results as well as have preceding research foundation.

The need for prognostic prediction after RC for UCB patients has induced the development of various postoperative models.^10^, ^11^, ^23^, ^24^ Among them, the International Bladder Cancer Nomogram Consortium (IBCNC) and the Bladder Cancer Research Consortium (BCRC) nomograms are well‐constructed and externally validated models.^10^, ^11^, ^12^, ^13^, ^25^, ^26^ The IBCNC model was developed to predict recurrence risk after RC for UCB patients based on international multicenter cohort. The nomogram incorporated seven variables, including age at RC, gender, pathologic T stage, histologic subtype, histologic grade, lymph node status, and time from diagnosis to RC. Meanwhile, the BCRC nomograms were designed to predict cancer‐specific, overall, and recurrence‐free survival after RC based on U.S. multicenter cohort. The models utilized eight variables, including lymphovascular invasion, which might not be routinely available in pathologic reports. They also included adjuvant radiotherapy, which may also not be commonly available since radiotherapy is only applied to a small fraction of patients with low grade of recommendation.^3^ In addition, both the IBCNC and the BCRC nomograms might not be generally applicative because: (a) the institutional cohorts from academic centers does not necessarily represent larger populations; (b) the number of included variables and the lack of access tools might increase calculation burden. Another prognostic model in recent years is the Cancer of the Bladder Risk Assessment (COBRA) score based on SEER population.^15^ The model served as a risk‐stratification tool which incorporated age, T stage, and lymph node density. However, it did not consider important information such as other aforementioned factors. Besides, the calculation process to obtain risk scores and referring to the outcome table might be an obstacle for potential users. The C‐indices of our nomogram were also larger than this model in both the development cohort (0.718 vs. 0.712) and validation cohort (0.707 vs. 0.705). In contrast, our nomogram utilizes conveniently available and necessary variables based on the largest population to date. Furthermore, a web tool is provided for easy access of the predictive model.

Our study has certain limitations to note. First, as the study is based on the SEER database, there is a lack of potential important factors, such as preoperative laboratory results, lymphovascular invasion, surgical margin status, comorbid conditions, and socioeconomic status. Second, we excluded patients with unknown histologic grade, AJCC stage, T stage, or N stage, which might introduce selection bias despite the small fraction. However, the large number of patients and the population‐based design could strengthen our model and reduce potential confounding impact. Finally, the results should be taken with caution due to the study's retrospective nature. Therefore, large prospective clinical trials for external validation are needed. Once it is further validated, our model could provide a foundation for future improved predictive tool incorporating potential multi‐omics profiles.

## 5 CONCLUSIONS

This large population‐based study revealed several demographic, clinicopathologic factors, and therapeutic features that were significantly associated with survival outcome of UCB patients after RC. We established and validated a prognostic nomogram to better predict 3‐ and 5‐year CSS probabilities than the AJCC stage for these patients. In addition, we developed a web tool for easy access to and improved utility of our model. This novel instrument may help clinicians in patient counseling, follow‐up scheduling, and potential clinical trial design for UCB patients who underwent RC. Still, large prospective clinical studies for external validation are needed.

## CONFLICT OF INTEREST

The authors declare that they have no conflict of interest.

## AUTHOR CONTRIBUTIONS

PH and ZQY conceptualized and supervised the conduct of the study; YJB and MYL conducted data collection and statistical analysis; ZQY and XH prepared the manuscript for submission; all authors reviewed and critiqued the manuscript for content.

## Data Availability

The data that support the findings of this study are available at the Surveillance, Epidemiology, and End Results database (https://seer.cancer.gov).
